# The passing of the Romanian Torch

**DOI:** 10.22336/rjo.2022.1

**Published:** 2022

**Authors:** Florian Baltă

**Affiliations:** * MD, PhD, FEBO, President of the Romanian Society of Ophthalmology

I am deeply honored and truly grateful to have been appointed the current President of Romanian Society of Ophthalmology. After a rather challenging period, facing the COVID-19 pandemic as a major health crisis, we hope 2022 will be the year to “right the ship” for both ophthalmologists and patients. 

Taking over the reins of the Romanian Society of Ophthalmology, a professional association with consistent activity and good reputation, we are keen on continuing the sterling work of our predecessors in promoting excellence in science, research, and education. As Ophthalmology continues to develop rapidly on all fronts, the Romanian Society of Ophthalmology has ambitious plans to keep the pace with the growing needs of its members.

The annual congress of Romanian Society of Ophthalmology will continue to be the beating heart of the Society. After one successful virtual congress in 2021, the COVID-19 pandemic has durably changed delegates’ expectations in terms of conference experience, our thoughts being now focused on the hybrid future of these meetings.

In the current era of information abundance, Romanian Journal of Ophthalmology has remained a growing project, settled among our priorities. From its beginning, the journal has remained integrated with and managed by the Society, its purpose being to add value to membership. Given the expanding importance of research metrics, we will look for opportunities to innovate our editorial processes and to increase its standards. 

With the global spread of digital approach, our intention is to expand the Society’s digital offering, transforming the current website into a dynamic resource center, packed with information, educational resources, and selected news, tailored for students, residents, and practitioners. An even more ambitious plan is the one of implementing the complex digital transformation process of eye health related activities, aiming the National Registration System used by all health and commissioning authorities.

Another key initiative is to reinvigorate the guidelines programme for specific ophthalmological conditions in order to help our members make informed, evidence-based decisions about appropriate and performant patient care.

We are fortunate to have an excellent and active Board, which has created a strategic plan, whereas supplementary tips and suggestions are always welcomed. I am both honored and excited to support colleagues across the country write the next chapter in the remarkable history of Romanian Journal of Ophthalmology!


**Prof. Florian Baltă, MD, PhD, FEBO,**
**President of the Romanian Society of Ophthalmology**


